# Transition Metal Borides for All-in-One Radiation Shielding

**DOI:** 10.3390/ma16196496

**Published:** 2023-09-29

**Authors:** Celal Avcıoğlu, Suna Avcıoğlu

**Affiliations:** 1 Fachgebiet Keramische Werkstoffe/Chair of Advanced Ceramic Materials, Institute of Material Science and Technology, Faculty III Process Sciences, Technische Universität Berlin, Straße des 17, Juni 135, 10623 Berlin, Germany; 2 Department of Metallurgical and Materials Engineering, Faculty of Chemistry and Metallurgy, Yıldız Technical University, 34956 Istanbul, Turkey

**Keywords:** radiation shielding, mixed gamma-neutron radiation, transition metal borides, attenuation coefficient, simulation, Phy-X/PSD, NGCal, EpiXS

## Abstract

All-in-one radiation shielding is an emerging concept in developing new-generation radiation protection materials since various forms of ionizing radiation, such as neutrons and gamma rays, can occur simultaneously. In this study, we examine the ability of transition metal borides to attenuate both photon and particle radiation. Specifically, fourteen different transition metal borides (including inner transition metal borides) are selected for examination based on their thermodynamic stabilities, molecular weights, and neutron capture cross-sections of the elements they contain. Radiation shielding characteristics of the transition metal borides are computationally investigated using Phy-X/PSD, EpiXS and NGCal software. The gamma-ray shielding capabilities of the transition metal borides are evaluated in terms of the mass attenuation coefficient (*μ_m_*), the linear attenuation coefficient (*µ*), the effective atomic number (*Z_eff_*), the half-value layer (HVL), the tenth-value layer (TVL), and the mean free path (MFP). The mass and linear attenuation factors are identified for thermal and fast neutrons at energies of 0.025 eV and 4 MeV, respectively. Moreover, the fast neutron removal cross-sections (∑R) of the transition metal borides are calculated to assess their neutron shielding abilities. The results revealed that borides of transition metals with a high atomic number, such as Re, W, and Ta, possess outstanding gamma shielding performance. At 4 MeV photon energy, the half-value layers of ReB_2_ and WB_2_ compounds were found as 1.38 cm and 1.43 cm, respectively. Most notably, these HVL values are lower than the HVL value of toxic Pb (1.45 cm at 4 MeV), which is one of the conventional radiation shielding materials. On the other hand, SmB_6_ and DyB_6_ demonstrated exceptional neutron attenuation for thermal and fast neutrons due to the high neutron capture cross-sections of Sm, Dy, and B. The outcomes of this study reveal that transition metal borides can be suitable candidates for shielding against mixed neutron and gamma radiation.

## 1. Introduction

Ionizing radiation is a form of energy that has a wide range of practical uses in different fields, such as power generation, healthcare, agriculture, and research [1,2,3,4]. Nevertheless, exposure to ionizing radiation can have significantly negative impacts on human health because ionizing radiation is powerful enough to detach electrons from atoms [5]. Therefore, the use of effective shielding materials is crucial to minimize human exposure to ionizing radiation.

Boron-containing materials are widely used in radiation shielding applications due to boron’s high neutron capture cross-section. Boron has two stable isotopes, ^10^B and ^11^B. Natural occurrence rates of ^10^B and ^11^B isotopes are 19.1–20.3% and 79.7–80.9%, respectively [6,7]. Thermal neutron capture cross-section (3838 barns; 1 barn per nucleus = 1 × 10^−24^ cm^2^) of the ^10^B isotope is significantly higher than that of boron’s other isotopes [8]. Therefore, ^10^B enriched boron carbide pellets are used in control rods of nuclear reactors [9]. Borax-containing water is used in fuel storage ponds of boiling water-based nuclear reactors to draw heat away from the reactor core. Further, the aqueous solution containing sodium borax also prevents reactor corrosion. Boron-doped steel and concrete are used in the construction of nuclear power plants [8]. In addition to the nuclear energy industry, boron also finds its applications in other radiation-related fields, such as nuclear medicine, where boron is used in neutron capture therapy applications (BNCT) [10,11].

Recently, polymer matrix composites as radiation shields received a lot of attention due to their lightweight and flexibility [7,12,13,14,15,16,17,18,19]. Boron carbide is one of the favorable reinforcement materials for radiation-shielding polymer matrix composites because of its high ^10^B content. The influence of matrix type, filler ratio, and distribution in the matrix phase and reinforcement’s particle size on the composite’s shielding performance has been widely studied [6,12,20,21,22]. Boron carbide-reinforced polymer matrix composites show excellent neutron shielding ability. Nevertheless, they exhibit poor gamma and X-ray shielding performance due to the low molecular weight of boron carbide [7,23,24]. 

Elements with high atomic numbers tend to provide effective shielding against high-energy radiation such as X-rays and gamma-rays. Indeed, lead, with a high density (11.5 g/cm^3^) and atomic number (Z = 82), is a traditional material used for gamma-ray protection, especially in nuclear power plants, medical diagnosis and treatment centers. However, due to its high toxicity, lead has to be covered with structural materials such as concrete, which increases the thickness of the shielding material [25]. Therefore, the use of lead in nuclear shielding applications is reducing, and the plates made of W, Al, Fe, and Cu metals are emerging alternatives. In concrete structures reinforced with metal plates, metal plates provide gamma protection, while concrete provides neutron protection with its high hydrogen and oxygen content. There are also cases where concrete is enriched with boron-containing elements for advanced neutron protection [26]. However, heavy materials such as lead, concrete, or metal plates are not suitable materials for every application. Especially in agriculture, gamma and X-rays are used for pest control, improving soil and water quality, and promoting plant growth. Therefore, unlike heavy concrete and metal plates, lighter materials with even optical transmittance are required [27]. Materials that are expected to have a radiation-absorbing effect as well as optical transmittance, are also needed in spare parts of nuclear medicine and various characterization devices [28]. Intensive efforts have been made in developing new generation glass and glass-ceramic materials, especially for such applications [29]. The effectiveness of various oxide compounds, including ZnO, CuO, Dy_2_O_3_, Al_2_O_3_, V_2_O_5_, SiO_2_, CdO, SrO, Bi_2_O_3_, CoO, and Nd_2_O_3_, as gamma-shielding additives in glass compositions has been intensively investigated due to their high molecular weight and ability to participate in the glass network structure [28,30,31,32,33,34,35,36,37].

Most materials used in the nuclear shielding industry today only provide good protection against either particle or photon type of radiation. Multi-layer radiation shields are commonly used to shield against both particle and photon radiation. In these multi-layer shields, one-layer shields against neutrons, and the other layer attenuates the gamma rays [38,39,40]. Nevertheless, stacking multiple layers results in thick, high-weight, and costly structures. 

To address these issues, new shielding materials with good neutron and gamma-ray attenuation capacities should be explored. Transition metal borides are a class of materials with fascinating properties such as superconductivity, high hardness, high melting temperatures, and ultra incompressibility [41]. Transition metal borides show high stoichiometric and structural flexibility (M_2_B, MB, MB_4_, MB_6_, M_3_B_2_, MB_2_, M_2_B_4_, etc.). Transition metal borides can be classified according to their boron-to-metal elemental ratio. Typically, boron-rich transition metal borides display a B:M ratio of at least 4:1 whereas compounds with lower boron-to-metal ratio are known as metal-rich borides. Borides containing group 4 and 5 transition metals are ultra-refractory materials due to their melting temperatures higher than 3000 °C and their ability to maintain their hardness up to 2000 °C [42]. Borides also exhibit high strength, chemical stability, thermal conductivity, electrical conductivity, wear and corrosion resistance [43]. The discovery of the superconductivity of magnesium diboride (MgB2) led researchers to investigate borides with a similar crystal structure as MgB2 and increased the interest in metal borides. It has been reported that the planar structure of hexagonally stacked boron atoms has a great influence on the superconductivity in MB2 [44]. Recently, borides of Nb, Mo, W, and Re were also found to display superconductivity.

Transition metal borides are the combination of high-atomic-weight elements and boron, which have high neutron capture cross-section. Furthermore, there are elements with a very large neutron absorption cross-sectional area among the f-block elements, which are a subset of transition metals and are frequently referred to as lanthanides, rare-earth elements, or inner transition metals. Therefore, it is anticipated that transition metal borides may possess good attenuation capacity for both particle and photon radiations. In this contribution, for the first time in the literature, the radiation shielding performance of transition metal boride compounds is comparatively investigated. 

## 2. Materials and Methods

### 2.1. Compound Selection

**Figure 1:**
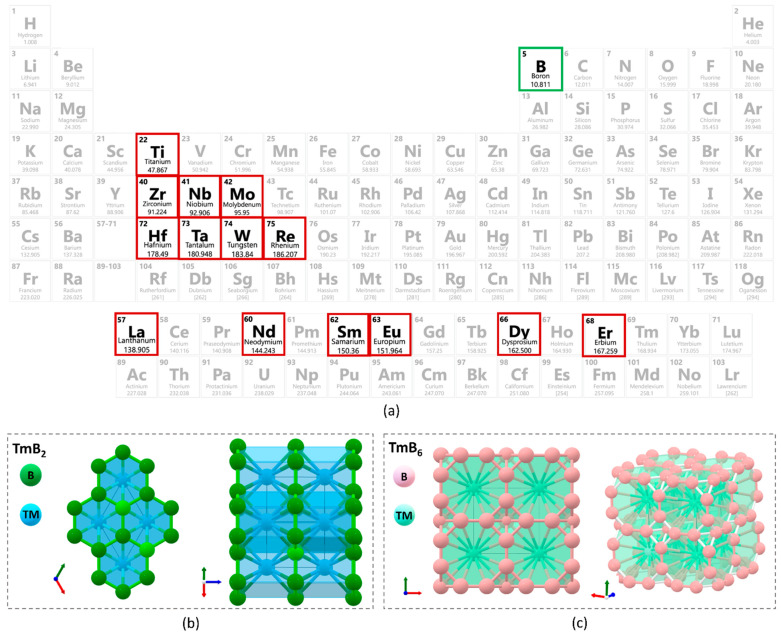
(**a**) Selected elements, crystal structures of (**b**) di-boride and (**c**) hexa-boride [45].

Two main parameters were considered to design an all-in-one radiation shielding compound. The compound should contain at least one high atomic number non-toxic element to achieve effective gamma and X-ray radiation shielding. The second criterion is the presence of at least one element with a high neutron absorption cross-section in the compound. Transition metal borides can be formed in a wide range of stoichiometric ratios and fulfill both requirements. To evaluate the shielding performance of transition metal borides, a wide range of elements with moderate to high atomic numbers (Z) were selected, and selected elements were emphasized in the periodic table in Figure 1a. Only thermodynamically stable compounds were selected. Borides of group 3 inner transition metals can form in hexa-boride (TMB_6_) with cubic crystal structure and Pm3¯m symmetry [45]. In a hexa-boride crystal structure, TM is bonded in a 24-coordinate geometry to twenty-four equivalent B atoms. TM–B bond lengths are in the range of 3.05 (for LaB_6_) -2.99 (for LuB_6_) Å. B is bonded in a 5-coordinate geometry to four equivalent TM and five equivalent B atoms. There is one short and four long B–B bonds. The length of short B-B bonds varies in the range of 1.66 (for LaB_6_) to 1.62 Å (for LuB_6_). Long B-B bonds vary from 1.76 (for LaB_6_) to 1.74 (for LuB_6_) [45,46]. On the other hand, di-boride (TMB_2_) stoichiometry with hexagonal crystal structure and P6/mmm symmetry is thermodynamically more stable for groups 4, 5, and 6 transition metal borides [47]. TM is bonded to twelve equivalent B^3−^ atoms to form a mixture of edge and face-sharing TMB_12_ cuboctahedra. All B–B bond lengths are the same, and W–B bond lengths vary in a wide range. The schematic drawings of crystal structures of di-borides and hexa-borides are presented in Figure 1b,c. Further details of transition metal borides, such as electronic state, phonon dispersion, diffraction patterns, aqueous stability, charge density, and other properties, can be found in the Materials Explorer application, which is shared openly in the public domain [48]. Therefore, hexa-borides of group 3 transition metals and di-borides of groups 4, 5, and 6 transition metal borides were selected to investigate and presented in Table 1.

### 2.2. Theoretical Calculations

Theoretical simulations were conducted using PHY-X/PSD, NGCal and EpiXS software, which are freely available in the public domains for academic use [49,50,51,52,53]. The density values of the investigated compounds listed in Table 1 were used for calculations. The calculations were conducted based on the molar ratios of boron and transition metals in each compound. The ratio of boron to transition metal in di-boride compounds is 2:1, and boron to inner transition metal in hexa-borides is 6:1. 

Firstly, the linear attenuation coefficients (LAC) and mass attenuation coefficients (MAC) were investigated. For a particular target, the LAC expresses the interaction chance between gamma rays and the target’s per unit thickness. It can be defined by Lambert–Beer law, as shown in Equation (1).

*I* = *I*_0_*e*^−*µx*^ (1)

*I*_0_ and *I* represent the gamma beam’s initial and attenuated intensity values. *x* is the target’s thickness in cm, and *µ* is the LAC (cm^−1^). The mass attenuation coefficient (*µ_m_*; cm^2^/g) can be obtained by dividing the LAC by the target’s density (*ρ*), equivalent to Equation (2).

*µ_m_* = (*µ*/*ρ*) = *ln* (*I*_0_/*I*)/*ρx* (2)

The half-value layer (HVL; cm) and the tenth-value layer (TVL; cm) are commonly employed parameters for describing the shielding performance of a target material. HVL and TVL specify the needed thicknesses to obtain a 50% and 90% reduction in the intensity of the initial radiation beam. Both of these values can be obtained from the linear attenuation coefficient (*μ*) using Equations (3) and (4).

HVL = *ln* (2)/*µ* (3)

TVL = *ln* (10)/*µ* (4)

Since the neutrons do not have any charge, they can pass through the electron shell of an atom and interact with its nucleus by multiple mechanisms such as scattering, nuclear fission, and neutron capture. This phenomenon complicates the determination of neutron removal cross-section in contrast to gamma photons. Nevertheless, calculating a fast neutron removal cross-section by using Equation (5) is an effective approach to gaining knowledge about the neutron shielding capability of matter. *∑_R_* is the total neutron removal cross-section, *ρ_i_* is the partial density, and (*∑_R_*/*ρ*)*_i_* (cm^2^/g) is the *i*th constituent’s mass removal cross-section.

∑*_R_* = ∑*ρ_i_* (∑*_R_*/*ρ*)*_i_* (5)

## 3. Results and Discussion

**Figure 2:**
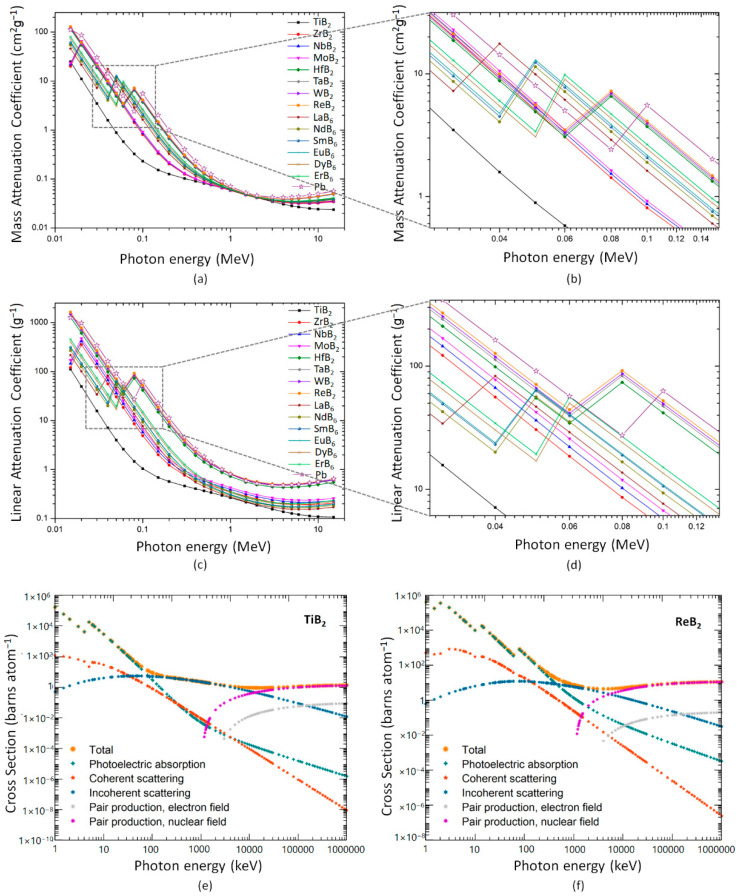
The variation in (**a**,**b**) the mass attenuation coefficient and (**c**,**d**) the linear attenuation coefficient with the incident photon energy for the transition metal borides and Pb. The total cross-section variations in (**e**) TiB2 and (**f**) ReB2 with the incident photon energy.

The mass attenuation coefficient describes the material’s radiation attenuation ability, and a higher coefficient implies a greater ability for gamma-ray shielding. Figure 2a,b demonstrates the relation between the mass attenuation coefficient of the composites and incident photon energy. The trend for the mass attenuation coefficient is similar for all transition metal borides, and the lowest tested energy yields individually the greatest mass attenuation coefficient values. Further, the attenuation coefficients of all investigated transition metal borides decrease rapidly with the increase in photon energy up to 0.1 MeV. Photoelectric absorption is the most prominent interaction mechanism for low-energy photons, and it depends highly on the atomic number of elements. Indeed, the likelihood of the photoelectric effect rises with increasing atomic number of the elements in the attenuator [54]. Therefore, in the low photon energy region up to 0.1 MeV, the mass attenuation coefficient values of the transition metal borides shows direct correlation with the atomic number of the transition metals and follows the order of ReB_2_ > WB_2_ > TaB_2_ > HfB_2_ > ErB_2_ > DyB_6_> EuB_6_ > SmB_6_ > NdB_6_> LaB_6_ > MoB_2_ > NbB_2_ > ZrB_2_ > TiB_2_. This suggests that the likelihood of atoms of ReB_2_ interacting with photons is more than that of other transition metal borides. Further, for all compounds except TiB_2_, an abrupt rise in the mass attenuation coefficient values at approximately 0.04–0.08 MeV was observed (Figure 2b). A photon is absorbed during the photoelectric absorption phenomenon, and an electron is ejected from the target atom. The energy of the absorbed photon is used to remove the electron from its shell, and the electron carries away all the remaining energy. However, an electron from a particular shell cannot be ejected if the photon energy falls below the binding energy of that shell. Since the K-shell electrons, which are the most tightly bound, require high energy to get ejected, characteristic “absorption edges” appear on attenuation coefficient variation versus photon energy plots [55]. So, the observed sudden rises in attenuation coefficient plots are because of the K-absorption edges of the transition metals. Since the titanium’s K-absorption edge is at 0.004966 MeV and the plots given in Figure 2a–d start from 0.01 MeV, it could not be seen in these plots. Nevertheless, the K-absorption edge of TiB_2_ is visible in Figure 2e [56].

In the intermediate energy level between 0.1 MeV and 3 MeV, the reduction in mass attenuation coefficients is slowed down for all compounds. In this region, Compton scattering is the dominant interaction mechanism, and it does not heavily depend on the atomic number of the elements. Therefore, the difference between the mass attenuation coefficient values of the investigated transition metal borides is markedly reduced. In the following energy region (beyond 3 MeV), the pair production phenomenon becomes dominant over Compton scattering. Unlike Compton scattering, the pair production cross-section is proportional to the squared atomic number Z^2^ of the scattering nucleus [57]. Due to this reason, the mass attenuation coefficient of the transition metal borides slowly increases above the photon energy range of 3 MeV.

We remark that the trends for linear and mass attenuation coefficients are expectedly similar, as seen in Figure 2c. Nevertheless, unlike the mass attenuation coefficient, the density influences the linear attenuation coefficient [58]. Therefore, the difference between the linear attenuation coefficient values of the transition metal borides is higher than their mass attenuation coefficient values (Figure 2b,d). Furthermore, the linear and mass attenuation coefficient variations in ReB_2_, WB_2_, and TaB_2_ are competitive with Pb in the selected energy region (Figure 2a–d).

The attenuation coefficient is the most crucial factor determining how gamma radiation penetrates and diffuses through extended media. The quantity of the attenuation coefficient depends on the photon energy E and the atomic number Z of the medium. Since the attenuation coefficient is proportional to the total photon interaction cross-section, the sum of the cross-sections for all the elementary scattering and absorption processes must be considered. The total cross-section can be considered as a sum of the photoelectric absorption, Compton collision, and pair production cross-sections per atom of a compound. The total cross-section variations in TiB_2_ and ReB_2_, which are the lowest and highest-performing compounds against photons, are presented separately in Figure 2e and Figure 2f, respectively. The dominant regions for photoelectric absorption, Compton collision, and pair production are seen in these figures. Most notably, the incoherent scattering dominates the larger photon energy range for the TiB_2_ compound in contrast to the ReB_2_. Because of Re’s higher Z value, ReB_2_ exhibits larger photoelectric absorption and pair production cross-sections and provides better shielding than TiB_2_.

**Figure 3:**
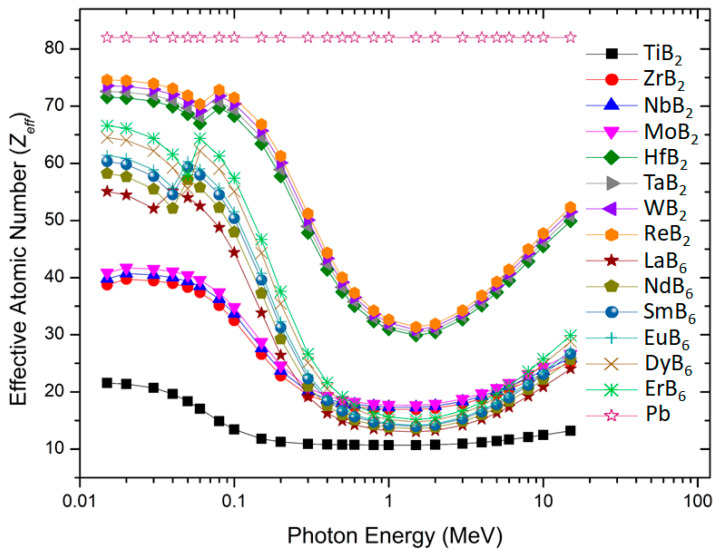
Variation in effective atomic numbers of the transition metal borides in the photon energy range of 0.015–15 MeV.

*Z_eff_* of the investigated transition metal borides against incident photon energies are illustrated in Figure 3. Generally, a material with a higher *Z_eff_* provides more targets to collide with protons than a material with a lower *Z_eff_*; hence, it experiences more interactions with photons. Therefore, a material with a high *Z_eff_* value is preferred for nuclear shielding applications. It is now well established that the compounds containing heavy atoms possess high *Z_eff_* values. Indeed, ReB_2_, WB_2,_ TaB_2,_ and HfB_2_ containing heavy transition metals exhibit comparatively higher *Z_eff_* values than other samples against all photon energies. 

On the other hand, the trend observed in *Z_eff_* is similar to the mass attenuation coefficient, *Z_eff_* of all samples first decreases then increases with the increase in photon energy. This is because of the dominating photoelectric effect, Compton scattering, and pair production in different energy zones. The *Z_eff_* values of compounds lie between 12 and 74. The highest *Z_eff_* values for all samples were obtained at 0.02 MeV due to the photoelectric absorption, and those are 74, 73, 72, and 71 for ReB_2_, WB_2,_ TaB_2,_ and HfB_2_, respectively. Contrarily, the lowest *Z_eff_* values occurred in the intermediate energy region where Compton scattering is dominant (0.3–4 MeV). Although the pair production event requires a minimum energy of 1.022 MeV, the dominance of this event usually begins after photon energies of 4 MeV. Therefore, a slight increase in the *Z_eff_* values beyond 4 MeV can be ascribed to the pair formation event. 

The effective atomic number variation of Pb against the photon energy is also seen in Figure 3. In contrast to the investigated boride compounds, the effective atomic number of Pb is almost constant against the whole selected energy region. The linear *Z_eff_* value variation indicates the stability of the target materials’ shielding performance with increased photon energy [50]. The observed high *Z_eff_* value change in boride compounds is because of the differences between the atomic number of boron and transition metal elements.

**Figure 4:**
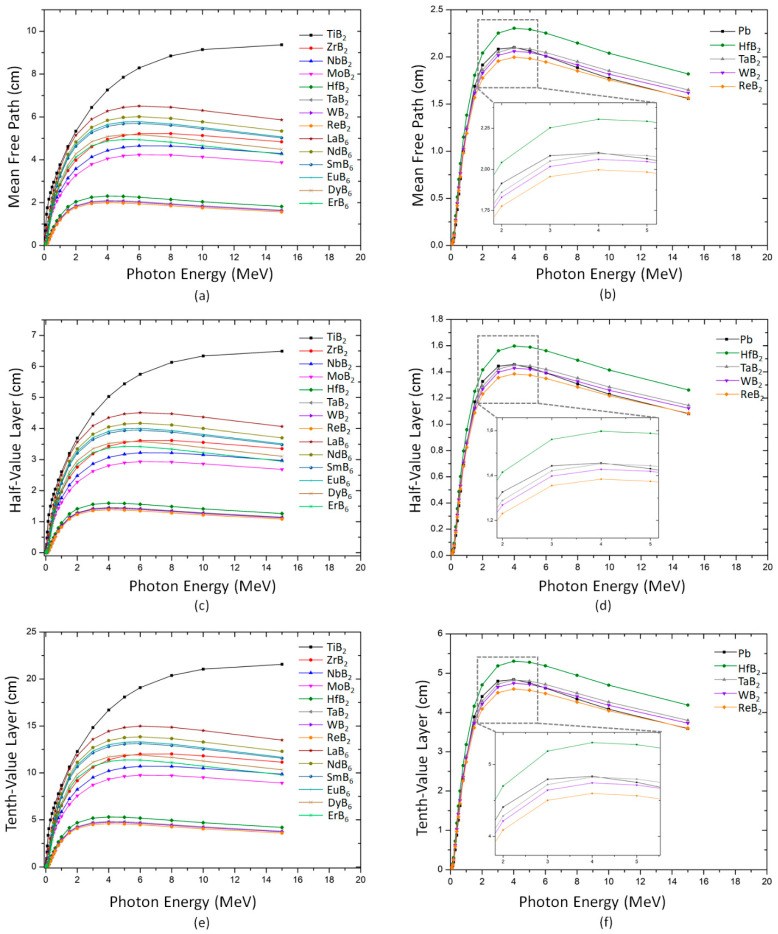
The variation in (**a**) the mean free path, (**c**) the half-value layer, and (**e**) the tenth-value layer with the photon energy for the transition metal borides. Comparison of (**b**) the mean free path, (**d**) the half-value layer and (**f**) the tenth-value layer of selected transition metal borides with Pb.

The mean free path, the half-value layer, and the tenth-value layer are important shielding parameters that can be used to determine the material’s amount and cost for providing sufficient radiation shielding. The mean free path is the average distance a photon travels in the radiation shield before it experiences a change in its energy or direction. On the other hand, the value layer and the tenth-value layer refer to the material thickness to lower the intensity of incident radiation by half and 90%, respectively. Therefore, the lower mean free path, half-value layer, and tenth-value layer values of a shielding material correspond to greater attenuating ability. The mean free path of the investigated transition metal borides for energies up to 15 MeV is given in Figure 4a. Obviously, the mean free path of the transition metal borides is low for low-energy photons. Nevertheless, the mean free path of transition metal borides demonstrates an increasing trend with the energy increment up to approximately 4 MeV. Then, it starts to decrease above the aforementioned energy due to the pair production effect. Indeed, the highest mean free path values for all transition metal borides are observed at approximately 4 MeV. Notably, the estimated mean free path of ReB_2_, WB_2,_ TaB_2,_ and HfB_2_ at all tested energies are significantly lower than other transition metal borides, which can be ascribed to the higher density of these compounds as compared to the other investigated transition metal borides. Meanwhile, TiB_2_ possesses the highest mean free path values at all tested photon energies due to the low atomic number of Ti. 

On the other hand, a similar trend to that of the mean free path was observed in the variation in both the half-value layer and the tenth-value layer with the incident energy. Indeed, ReB_2_, WB_2_, TaB_2_, and HfB_2_ demonstrated significantly less half-value layer and tenth-value layer values, as seen in Figure 4c,e. In these plots, ReB_2_ has the lowest half-value layer and tenth-value layer values. At 4 MeV, the half-value layer and the tenth-value layer of ReB_2_ reach their maximum, with 1.38 cm and 4.59 cm, respectively. Note that despite its high toxicity, lead is the most commonly used material in nuclear shielding due to its excellent gamma shielding ability. Therefore, the mean free path, half-value layer, and tenth-value layer values of ReB_2_, WB_2,_ TaB_2,_ and HfB_2_ are compared with those of lead, and the results are presented in Figure 3b,d,f. It can be seen that ReB_2_, WB_2,_ and TaB_2_ have better radiation shielding capacities relative to lead in the selected energy regions. It is notable to mention that Re is one of the rarest metals with an approximate abundance of 1 μg kg^−1^ [59]. On the contrary, the earth’s crust contains 10^3^ times higher W and Ta than Re. The results indicate that WB_2_ and TaB_2_ performed considerably similar shielding performance with ReB_2_. So, to fabricate all-in-one radiation shielding materials, choosing WB_2_ and TaB_2_ over ReB_2_ would be more cost-effective and sustainable.

**Figure 5:**
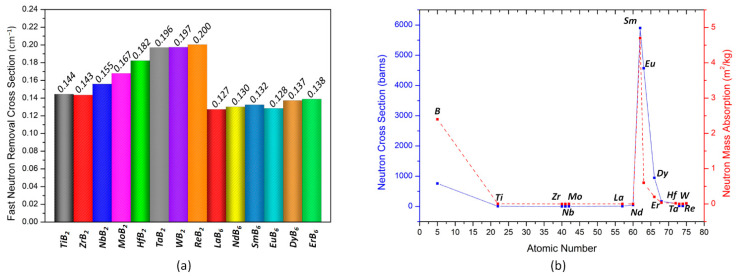
The fast neutron removal cross-sections (**a**) of the investigated transition metal borides and neutron capture cross-sections (**b**) of elements.

In addition to the gamma shielding characteristics, the fast neutron removal cross-section parameter of the transition metal borides was also investigated to assess their total macroscopic cross-sections for fast neutrons. Note that boron is a great neutron absorber due to its high neutron cross-section, as seen in Figure 5b. Therefore, boron-containing materials are commonly used as neutron shields. The fast neutron removal cross-section values of the investigated transition metal borides vary in the range of 0.127–0.2 cm^−1,^ as seen in Figure 5a. Since the density of the compound and the weight fractions of the elements it contains are also important factors in calculating the fast neutron removal cross-section, the decreasing fast neutron removal cross-section order is ReB_2_ > WB_2_ > TaB_2_ > HfB_2_ > MoB_2_ > NbB_2_ > TiB_2_ ZrB_2_ > ErB_6_ > DyB_6_ > SmB_6_ > NdB_6_ > LaB_6_ > EuB_6_. Although the neutron capture cross-section of Sm is the highest, its contribution to the fast neutron removal cross-section is rather small. This is because of the relatively lower density of SmB_6_ and weight fraction of Sm in SmB_6._ It is also worth mentioning that several transition metal borides investigated in this study outperform many extensively used neutron shielding materials. The calculated fast neutron removal cross-section values of the ReB_2_, WB_2_, TaB_2_, HfB_2_, MoB_2_, NbB_2_, ZrB_2_, and TiB_2_ are significantly higher than those of lead (0.118 cm^−1^), B_4_C (0.141 cm^−1^), NiO and PbO added borate glasses (0.111 cm^−1^), concrete (0.094 cm^−1^), graphite (0.077 cm^−1^), and paraffin (0.077 cm^−1^) [30,35,60]. 

**Figure 6:**
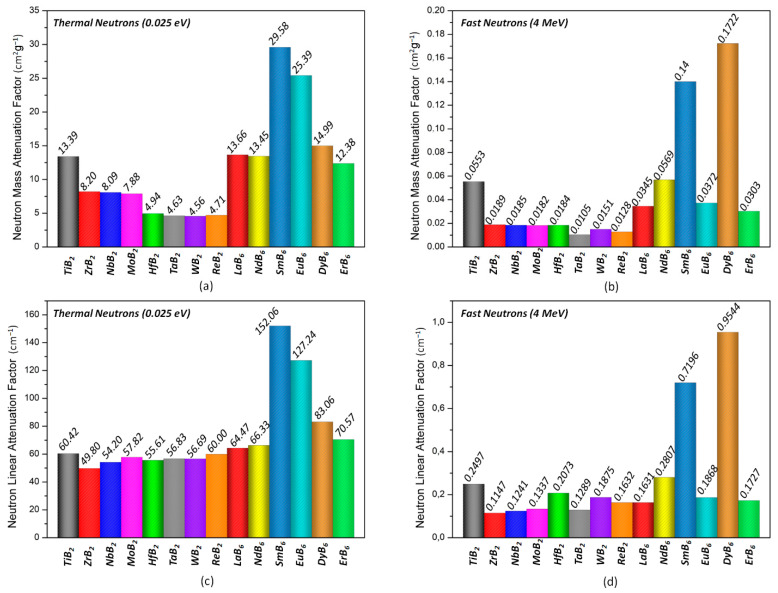
The mass (**a**,**b**) and linear (**c**,**d**) attenuation factors of selected compounds for thermal and fast neutrons.

Further the fast neutron removal cross-sections of transition metal borides, the mass and linear attenuation factors for thermal (0.025 eV) and fast (4 MeV) neutrons at specific energies were also investigated (Figure 6). The results provided by NGCal software were found to be consistent with the neutron capture cross-section of elements that constitute the transition metal boride compounds shown in Figure 5b. SmB_6_, EuB_6_, and DyB_6_ outperformed the rest of the transition metal borides on attenuating thermal neutrons with 0.025 eV energy, as seen in Figure 6a,c. On the other hand, for fast neutrons (4 MeV), DyB_6_ showed the highest neutron attenuation Figure 6b,d. Due to dysprosium’s larger neutron capture cross section at 4 MeV, DyB_6_ even exceeded SmB_6_.

The mass and linear neutron attenuation factors of widely used B_4_C were also calculated to compare with selected transition metal borides (Table 2). For fast neutrons at 4 MeV energy, both the mass and linear neutron attenuation factors of B_4_C were found to be lower than SmB_6_ and DyB_6_. The mass neutron attenuation factor of B_4_C with 33.51172 at a neutron energy of 0.025 eV is slightly higher than SmB_6_. Nevertheless, it decreases to 0.07678 for fast neutrons. 

It can be concluded that transition metal borides provide excellent particle and photon shielding simultaneously, and they have the potential to surpass widely used radiation shielding materials Pb and B_4_C. Nevertheless, further experimental tests are required to verify the theoretical model’s accuracy and the transition metal borides’ true potential.

## 4. Conclusions

In this study, photon and particle radiation shielding capacities of 14 different transition metal borides were evaluated, and the results were compared with commonly used radiation shielding materials. A thorough examination of the MAC, LAC, MFP, HVL, TVL, *Z_eff_*, and FNRCS parameters demonstrated that transition metal borides possess excellent gamma and neutron radiation attenuation properties. MAC values of the transition metal borides exhibit a strong correlation with the atomic number of the transition metals and follow the order of ReB_2_ > WB_2_ > TaB_2_ > HfB_2_ > ErB_6_ > DyB_6_ > EuB_6_ > SmB_6_ > NdB_6_ > LaB_6_ > MoB_2_ > NbB_2_ > ZrB_2_ > TiB_2_. Meanwhile, MFP values of ReB_2_, WB_2,_ and TaB_2_ at 4 MeV are 1,99, 2.06, and 2.09, respectively. These results indicate that these three materials have superior gamma-ray attenuation capability than lead, which is the most commonly used radiation shielding material in nuclear applications. 

On the other hand, the FNRCS (∑R) of the transition metal borides depended on the density of the compounds and was found to increase as the density of the compounds increased. Seven transition metal borides, including ReB_2_ (0.2 cm^−1^), WB_2_ (0.197 cm^−1^), TaB_2_ (0.196 cm^−1^), HfB_2_ (0.182 cm^−1^), MoB_2_ (0.167 cm^−1^), NbB_2_ (0.155 cm^−1^), ZrB_2_ (0.143 cm^−1^)_,_ and TiB_2_ (0.144 cm^−1^), were found to have better fast neutron removal cross-sections than Pb (0.118 cm^−1^), B_4_C (0.141 cm^−1^), borate glasses (0.111 cm^−1^), concrete (0.094 cm^−1^), and graphite (0.077 cm^−1^). The ReB_2_ has the highest fast neutron removal cross-section among the investigated transition metal borides. The transition metal boride’s mass and linear neutron attenuation factors strongly correlate with the neutron capture cross-section of constituted elements. The borides of all rare-earth transition metals showed better neutron attenuation for thermal neutrons with 0.025 eV energy than the rest. For thermal neutrons, higher neutron capture cross-sections of Sm, Eu, and Dy led to the best neutron attenuation performance for SmB_6_, EuB_6_, and DyB_6_ compounds. On the other hand, for fast neutrons with 4 MeV energy, DyB_6_ outperformed. Most notably, SmB_6_ and DyB_6_ were found to have superior neutron attenuation factors than B_4_C. The cost is also an important factor in designing sustainable radiation shielding materials. The low abundance of some rare earth elements and Re can increase the cost of their boride compounds. Nevertheless, the comparable price/performance ratio of WB_2_, TaB_2_, and SmB_6_ with B_4_C and nontoxicity over Pb make these compounds potential candidates for future developments in nuclear protection. Consequently, transition metal borides, especially ReB_2_, WB_2_, TaB_2_, SmB_6_, and DyB_6_, show not only great gamma-ray shielding but also desirable neutron attenuation characteristics and can be excellent candidates for nuclear safety applications.

## Figures and Tables

**Table 1:** Theoretical density values of the investigated compounds [48].

	Di-Borides							
	TiB_2_	ZrB_2_	NbB_2_	MoB_2_	HfB_2_	TaB_2_	WB_2_	ReB_2_
Density (g/cm^3^)	4.52	6.07	6.78	7.33	11.24	12.27	12.42	12.62
	**Hexa-Borides**							
	LaB_6_	NdB_6_	SmB_6_	EuB_6_	DyB_6_	ErB_6_		
Density (g/cm^3^)	4.72	4.93	5.14	5.01	5.54	5.70		

**Table 2:** The comparison of the neutron attenuation factors of SmB_6_ and DyB_6_ with B_4_C.

	The Mass Attenuation Factors		The Linear Attenuation Factors	
	Thermal Neutrons (0.025 eV)	Fast Neutrons (4 MeV)	Thermal Neutrons (0.025 eV)	Fast Neutrons (4 MeV)
B_4_C	33.51172	0.07678	84.44954	0.19349
SmB_6_	29.58439	0.14001	152.06374	0.71963
DyB_6_	14.99297	0.17228	70.57155	0.17271

## Data Availability

The data that support the findings of this study are available from the corresponding author upon reasonable request.
